# Supplementary material for: Access to, usage and clinical outcomes of, online postal sexually transmitted infection services: a scoping review
Source: Sex Transm Infect. 2022 Jun 14;98(7):528–35. doi: 10.1136/sextrans-2021-055376 (PMC9613868; doi:10.1136/sextrans-2021-055376)
Supplement: Supplementary data [file sextrans-2021-055376supp001.pdf]

Supplementary File

Supplemental File 1: Search Strategy (where applicable limited to publications in English language, 2010-2020).

Database	Medical Subject Headings	Keywords
Medline	exp Sexually Transmitted Diseases, exp Sexual Health, exp Telemedicine, exp Internet, exp Health Services Accessibility, exp Healthcare Disparities, exp Socioeconomic Factors, exp Minority Groups, exp Social Marginalization, exp United Kingdom<br><br>**NOT** exp Africa, exp americas, exp antarctic regions, exp arctic regions, exp asia, exp oceania	"sexually transmitted disease*", "sexually transmitted infection*", sti, stis, std, stds, venereal, "sexual health*", chlamydia, gonorrhoea, gonorrhoea, syphilis, "herpes genitalis", hiv, "human immunodeficiency virus*", "acquired immunodeficiency syndrome*", aids, "acute retroviral syndrome*", ars, "hepatitis B", "hepatitis C" telemedicine*, telehealth*, "mobile health*", mhealth*, ehealth*, internet, "digital service*", "digital intervention*", "digital technolog*", "self sampl*", "self test*", "home test*", "test* kit*", "self collect*", "home base*", "web-base*", "self-swab*", "home swab*", "mobile technolog*", "mobile application*", app, apps, "social medi*", "cell phone*", cellphone*, "mobile phone*", "mobile telephone*", "cellular phone*", smartphone*, "smart phone*", "mobile device*", "text messag*", texting, texted, sms, mms, "multimedia messag*", "short messag*", "computers, handheld", "personal digital assistant", email*, "e-mail*", online, "digital health*", access*, disparit*, barrier*, availab*, inaccess*, unavailab*, socioeconomic*, minorit*, inequalit*, equalit*, inequit*, "equit", "marginaliz*", "marginalis*", "convenien*", "inconvenien*", "hard to reach", "national health service*", nhs* , gb, "g.b.", britain*, british*, uk, "u.k.", "united kingdom*", england*, “english”, "northern ireland*", "northern irish*", scotland*, scottish*, wales, “south wales”, welsh*<br><br>**NOT** “british columbia”, “new england”, “new south wales”, ((published or publication* or translat* or written or language* or speak* or literature or citation*) adj5 english)
EMBASE	exp sexually transmitted disease, exp sexual health, exp telemedicine, exp internet, exp health care access, exp health care disparity, exp socioeconomic, exp	"sexually transmitted disease*", "sexually transmitted infection*", sti, stis, std, stds, venereal, "sexual health*", chlamydia, gonorrhoea, gonorrhoea, syphilis, "herpes genitalis", hiv, "human immunodeficiency virus*", "acquired immunodeficiency syndrome*", aids, "acute retroviral syndrome*", ars, "hepatitis B", "hepatitis C" telemedicine*, telehealth*, "mobile health*", mhealth*, ehealth*, internet, "digital service*", "digital intervention*", "digital technolog*", "self sampl*", "self test*", "home test*", "test* kit*", "self collect*", "home base*", "web-base*", "self-swab*", "home swab*", "mobile technolog*", "mobile application*", app, apps, "social medi*", "cell phone*", cellphone*, "mobile phone*", "mobile telephone*", "cellular phone*",

	minority group, exp social exclusion, exp United Kingdom<br><br>**NOT** exp africa, exp americas, exp antarctic regions, exp arctic regions, exp asia, exp oceania	smartphone*, "smart phone*", "mobile device*", "text messag*", texting, texted, sms, mms, "multimedia messag*", "short messag*", "computers, handheld", "personal digital assistant", email*, "e-mail*", online, "digital health*", access*, disparit*, barrier*, availab*, inaccess*, unavailab*, socioeconomic*, minorit*, inequalit*, equalit*, inequit*, "equit", "marginaliz*", "marginalis*", "convenien*", "inconvenien*", "hard to reach", "national health service*", nhs* , gb, "g.b.", britain*, british*, uk, "u.k.", "united kingdom*", england*, "english", "northern ireland*", "northern irish*", scotland*, scottish*, wales, "south wales", welsh*,<br><br>**NOT** "british columbia", "new england", "new south wales", ((published or publication* or translat* or written or language* or speak* or literature or citation*) adj5 english)
PsycInfo<br><br>HMIC	exp sexually transmitted diseases, exp sexual health, exp telemedicine, exp internet, exp health care access, exp health disparities, exp socioeconomic status, exp minority groups	"sexually transmitted disease*", "sexually transmitted infection*", sti, stis, std, stds, venereal, "sexual health*", chlamydia, gonorrhoea, gonorrhoea, syphilis, "herpes genitalis", hiv, "human immunodeficiency virus*", "acquired immunodeficiency syndrome*", aids, "acute retroviral syndrome*", ars, "hepatitis B", "hepatitis C" telemedicine*, telehealth*, "mobile health*", mhealth*, ehealth*, internet, "digital service*", "digital intervention*", "digital technolog*", "self sampl*", "self test*", "home test*", "test* kit*", "self collect*", "home base*", "web-base*", "self-swab*", "home swab*", "mobile technolog*", "mobile application*", app, apps, "social medi*", "cell phone*", cellphone*, "mobile phone*", "mobile telephone*", "cellular phone*", smartphone*, "smart phone*", "mobile device*", "text messag*", texting, texted, sms, mms, "multimedia messag*", "short messag*", "computers, handheld", "personal digital assistant", email*, "e-mail*", online, "digital health*", access*, disparit*, barrier*, availab*, inaccess*, unavailab*, socioeconomic*, minorit*, inequalit*, equalit*, inequit*, "equit", "marginaliz*", "marginalis*", "convenien*", "inconvenien*", "hard to reach", "national health service*", nhs* , gb, "g.b.", britain*, british*, uk, "u.k.", "united kingdom*", england*, "northern ireland*", "northern irish*", scotland*, scottish*, wales, "south wales", welsh*<br><br>**NOT** "british columbia", "new england", "new south wales"
CINAHL Plus	(MH "Sexually Transmitted Diseases+"), (MM "Sexual Health"), (MH "Telemedicine+"), (MH "Internet+"), (MH "Health Services Accessibility+"), (MM "Healthcare Disparities"), (MH "Socioeconomic Factors+"),	"sexually transmitted disease*", "sexually transmitted infection*", sti, stis, std, stds, venereal, "sexual health*", chlamydia, gonorrhoea, gonorrhoea, syphilis, "herpes genitalis", hiv, "human immunodeficiency virus*", "acquired immunodeficiency syndrome*", aids, "acute retroviral syndrome*", ars, "hepatitis B", "hepatitis C" telemedicine*, telehealth*, "mobile health*", mhealth*, ehealth*, internet, "digital service*", "digital intervention*", "digital technolog*", "self sampl*", "self test*", "home test*", "test* kit*", "self collect*", "home base*", "web-base*", "self-swab*", "home swab*", "mobile technolog*", "mobile application*", app, apps, "social medi*", "cell phone*", cellphone*, "mobile phone*", "mobile telephone*", "cellular phone*", smartphone*, "smart phone*", "mobile device*", "text messag*", texting, texted, sms, mms, "multimedia messag*", "short messag*", "computers, handheld", "personal digital assistant", email*, "e-mail*", online,

	(MM "Minority Groups"), (MH “United Kingdom”)	"digital health*", access*, disparit*, barrier*, availab*, inaccess*, unavailab*, socioeconomic*, minorit*, inequalit*, equalit*, inequit*, "equit", "marginaliz*", "marginalis*", "convenien*", "inconvenien*", "hard to reach", "national health service*", nhs* , gb, "g.b.", britain*, british*, uk, "u.k.", "united kingdom*", england*, "northern ireland*", "northern irish*", scotland*, scottish*, wales, “south wales”, welsh*<br><br>**NOT** “british columbia”, “new england”, “new south wales”
Scopus<br><br>Open Grey<br><br>Web of Science	n/a	"sexually transmitted disease*", "sexually transmitted infection*", sti, stis, std, stds, venereal, "sexual health*", chlamydia, gonorrhoea, gonorrhoea, syphilis, "herpes genitalis", hiv, "human immunodeficiency virus*", "acquired immunodeficiency syndrome*", aids, "acute retroviral syndrome*", ars, "hepatitis B", "hepatitis C" telemedicine*, telehealth*, "mobile health*", mhealth*, ehealth*, internet, "digital service*", "digital intervention*", "digital technolog*", "self sampl*", "self test*", "home test*", "test* kit*", "self collect*", "home base*", "web-base*", "self-swab*", "home swab*", "mobile technolog*", "mobile application*", app, apps, "social medi*", "cell phone*", cellphone*, "mobile phone*", "mobile telephone*", "cellular phone*", smartphone*, "smart phone*", "mobile device*", "text messag*", texting, texted, sms, mms, "multimedia messag*", "short messag*", "computers, handheld", "personal digital assistant", email*, "e-mail*", online, "digital health*", access*, disparit*, barrier*, availab*, inaccess*, unavailab*, socioeconomic*, minorit*, inequalit*, equalit*, inequit*, "equit", "marginaliz*", "marginalis*", "convenien*", "inconvenien*", "hard to reach", "national health service*", nhs* , gb, "g.b.", britain*, british*, uk, "u.k.", "united kingdom*", england*, "northern ireland*", "northern irish*", scotland*, scottish*, wales, “south wales”, welsh*<br><br>**NOT** “british columbia”, “new england”, “new south wales”
Ethos	n/a	1. “sexually transmitted infection” AND test<br>2. “sexually transmitted infection” AND online<br>3. “sexually transmitted infection” AND access<br>4. “sexually transmitted infection” AND UK<br>5. “sexually transmitted disease” AND test<br>6. “sexually transmitted disease” AND online<br>7. “sexually transmitted disease” AND access<br>8. “sexually transmitted disease” AND UK

Supplemental File 2: Systematic Review Data Extraction Form (blank)

Systematic Review Data Extraction Form

Record no.	
Article citation	
Type:	
Completed by:	

Summary Notes

Study Description

Study aims/research questions	
Rationale (why did the study author believe the study could benefit the study population in this context?)	
Participants (number and description)	
Setting (geographic location, institutional setting, other place/space-related features)	
Summary of intervention	

Data Collection

What types of data are collected/managed/shared?	
How is this data collected/managed/shared? (describe the tool, if there is one)	
Data collection and sharing context (who is collecting data and who is it being shared with, how?)	

Study Design

Description of study design	
Theoretical framework for development and/or evaluation (describe, if there is one)	
Eligibility (inclusion/exclusion criteria)	
Control/comparison group	
Analyses conducted	
Outcome measures	

Study Findings (add more rows to elaborate on findings as needed)

Key descriptive statistics	
Clinical outcomes	
Other self-reported outcomes	
Acceptability	

Assessment & Impacts

Strengths and Limitations (as noted by authors)	
MMAT Assessment Results	
Key impacts and lessons learned	

Key Quotes (for thematic analysis, add more rows as needed)

Notes	Quote

Supplemental File 3: Demographics of Study Participants

Service	Author	Study population & number of participants	Gender	Age	Ethnicity	Sexual orientation	Index of deprivation
**eSexual Health Clinic (eSHC)**	Aicken et al. 2018 (37)	Telephone interviews with patients who had used the eSHC N=36	Female - 20<br>Male - 16	18-24yrs – 18<br>25-35yrs - 18	Asian - 3<br>Black - 7<br>Mixed - 4<br>White - 22	Heterosexual – 34<br>Unstated - 2	Unstated
	Estcourt et al. 2017 (38)	Recruited from GUM clinic - 116	Female - 74<br>Male - 42	Median 25yrs	White British – 37<br>White other – 29<br>Black – 17<br>Asian, mixed or other – 21<br>Unstated - 12	Same-sex partner in last 6 months - 1	Unstated
		Recruited from NCSP Checkurself - 105	Female - 60<br>Male - 45	Median 22yrs	White British – 67<br>White other – 5<br>Black – 12<br>Asian, mixed or other – 10<br>Unstated - 11	Same-sex partner in last 6 months – 3	Unstated
	Gibbs et al. 2018 (39)	1) Patients completing telephone survey - 152<br>2) Interviews (see Aicken et al. 2018)<br>3) Online survey - 331	Unstated	Unstated	Unstated	Unstated	Unstated
**Freetest.me**	Dolan et al. 2014 (40)	2988	Male: 33.7<br>Female:66.3	(Mean)<br>16-19yrs – 30.7<br>20-24yrs – 66.4<br>Unstated – 2.9	(Mean)<br>White – 86.5<br>Black – 1.4<br>Asian – 1.3<br>Other – 0.3<br>Mixed – 3.1	Unstated	(Mean)<br>IMD Score (SD) – 20.2 (14.13)

					Unstated – 7.3		
**Letstalkaboutit**	Gasmelsid et al. 2021 (34)	Before online testing 2847	Female 1561<br>Male 1285	<25 1949<br>25+ 898	Black and Minority Ethnic: 209<br>White: 2099	Non-heterosexual 120<br>Heterosexual 1336	Unstated, but reported as no difference between groups
		After online testing 2066 clinic	Female 1007<br>Male 891	<25 1334<br>25+ 732	Black and Minority Ethnic: 227<br>White: 1540	Non-heterosexual 51<br>Heterosexual 875	
		After online testing 775 online	Female 437<br>Male 210	<25 520<br>25+ 255	Black and Minority Ethnic: 33<br>White: 366	Non-heterosexaul 28<br>Heterosexual 213	
**NCSP**	Woodhall et al. 2012 (5)	Internet test kits requested (number of participants unstated) – 59719	Female: 38268	15yrs – 442<br>16-19yrs – 13099<br>20-24yrs – 24727	White – 32604<br>Black – 796<br>Asian – 747<br>Chinese – 74<br>Other – 91<br>Mixed – 1032<br>Unstated – 2924	Unstated	IMD quintile<br>1 (most deprived) – 6662<br>2 – 7965<br>3 – 7987<br>4 – 7341<br>5 – 7019<br>Unstated – 1294
			Male: 21451	15yrs – 237<br>16-19yrs – 6659<br>20-24yrs – 14555	White – 17925<br>Black – 499<br>Asian – 519<br>Chinese – 24<br>Other – 59<br>Mixed – 569<br>Unstated – 1855	Unstated	IMD quintile<br>1 (most deprived) – 3733<br>2 – 4276<br>3 – 4353<br>4 – 4093<br>5 – 4164<br>Unstated – 832
		GP tests (number of participants unstated) – 148619	Female: 109,187	15yrs – 3322<br>16-19yrs – 41698<br>20-24yrs – 64167	White – 67934<br>Black – 5851<br>Asian – 5464<br>Chinese – 721<br>Other – 540<br>Mixed – 2670<br>Unstated – 26007	Unstated	IMD quintile<br>1 (most deprived) – 26319<br>2 – 24489<br>3 – 18318<br>4 – 14620<br>5 – 15565<br>Unstated – 9876
			Male: 39432	15yrs – 901<br>16-19yrs – 14283<br>20-24yrs – 24248	White – 20976<br>Black – 2784<br>Asian – 4744<br>Chinese – 335	Unstated	IMD quintile<br>1 (most deprived) – 10837<br>2 – 9525<br>3 – 6186

					Other – 298<br>Mixed – 950<br>Unstated – 9345		4 – 4571<br>5 – 4495<br>Unstated – 3818
		SRH tests (number of participants unstated) – 202028	Female: 156,432	15yrs – 13463<br>16-19yrs – 82264<br>20-24yrs –60705	White – 97153<br>Black – 11886<br>Asian – 4127<br>Chinese – 510<br>Other – 733<br>Mixed – 6139<br>Unstated – 35884	Unstated	IMD quintile<br>1 (most deprived) – 50020<br>2 – 35086<br>3 – 24394<br>4 – 19514<br>5 – 15441<br>Unstated – 11977
			Male: 45596	15yrs – 3215<br>16-19yrs – 24866<br>20-24yrs – 17515	White – 26261<br>Black – 4002<br>Asian – 1488<br>Chinese – 75<br>Other – 194<br>Mixed – 1703<br>Unstated – 11783	Unstated	IMD quintile<br>1 (most deprived) – 15331<br>2 – 10053<br>3 – 6395<br>4 – 5523<br>5 – 3952<br>Unstated – 4342
**North East Essex PCT**	Bracebridge et al. 2012 (41)	People offered screening - 29917	Female: 14773<br>Male: 15136<br>Unstated: 8	17-18yrs – 3773<br>19yrs – 4512<br>20yrs – 4532<br>21yrs – 4643<br>22yrs – 4220<br>23yrs – 4099<br>24-25yrs – 4128<br>Unstated – 10	Unstated	Unstated	IMD quintile<br>1 (least deprived)* – 5857<br>2 – 6048<br>3 – 6004<br>4 – 5992<br>5 (most deprived)* – 6002<br>Unstated – 14
		People who completed screening - 3431	Female: 1951<br>Male: 1480	17-18yrs – 466<br>19yrs – 564<br>20yrs – 569<br>21yrs – 466<br>22yrs – 444<br>23yrs – 434<br>24-25yrs – 486	White – 2967<br>Other – 216<br>Unstated – 248	Unstated	IMD quintile<br>1 (least deprived)* – 389<br>2 – 802<br>3 – 872<br>4 – 687<br>5 (most deprived)* – 676<br>Unstated – 5

				Unstated – 2			
		People who tested positive for chlamydia - 152	Female: 85<br>Male: 67	17-18yrs – 18<br>19yrs – 21<br>20yrs – 20<br>21yrs – 20<br>22yrs – 32<br>23yrs – 21<br>24-25yrs – 20	White – 135<br>Other – 6<br>Unstated – 11	Unstated	IMD quintile<br>1 (least deprived)* – 11<br>2 – 39<br>3 – 26<br>4 – 42<br>5 (most deprived)* – 34
**Saving Lives**	Page et al. 2019 (42)	Users of Mini-tube - 275	Female – 166<br>Male – 106<br>Trans – 2<br>Unspecified – 0	Median 26, IQR 22, 31<br>Mean 28, 95% CI 27, 29	Any other mixed background – 2<br>Any other white background – 7<br>Bangladeshi – 1<br>Black African – 0<br>Black Caribbean - 0<br>Chinese - 0<br>Indian – 1<br>Unknown/Not specified – 3<br>White & Asian – 4<br>White and black African – 2<br>White and black Caribbean - 3<br>White British - 242<br>White Irish - 10	Heterosexual Male – 86<br>Heterosexual Female – 152<br>MSM - 20<br>WSW - 16<br>Unknown/not spec - 1	Unstated
		Users of dried blood spot - 275	Female – 94<br>Male – 181<br>Trans – 0<br>Unspecified – 0	Median 25, IQR 22, 30<br>Mean 28, 95% CI 27,29	Any other mixed background – 2<br>Any other white background – 5<br>Bangladeshi – 0<br>Black African – 1<br>Black Caribbean - 1<br>Chinese - 2<br>Indian – 0<br>Unknown/Not specified – 1<br>White & Asian – 3<br>White and black African – 0<br>White and black Caribbean - 1	Heterosexual Male – 66<br>Heterosexual Female – 167<br>MSM - 28<br>WSW - 14<br>Unknown/not spec - 0	Unstated

					White British - 253<br>White Irish - 6		
Sexual Health London	Day et al. 2020 (43)	250 reported recent sexual assault on online triage form	Female: 135<br>Male: 114<br>Trans or non-binary: 1	Mean 26<br>Range 18-55	African – 28<br>Asian – 24<br>Caribbean – 23<br>Other – 64<br>White – 111	Heterosexual men – 75<br>Heterosexual women – 105<br>MSM – 30<br>WSW – 2<br>Bisexual men – 9<br>Bisexual women – 28<br>Undetermined - 1	Unstated
	Day et al. 2021 (44)	118,825 registrants identifying as cisgender,	Cisgender F:66955<br>Cisgender M: 51870	Median age 27<br>Range 15-82	African – 5537<br>Asian – 5781<br>Caribbean – 6417<br>Other – 17,482<br>White – 39082	Unstated	Unstated
		504 registrants identifying as transgender, non-binary or 'other'	Trans men 76<br>Trans women 78<br>Non-binary/gender-fluid 17<br>Other 33	Median age 27<br>Range 16-82	African – 14<br>Asian – 21<br>Caribbean – 12<br>Other - 100<br>White - 145	Unstated	Unstated
	Day et al. 2020 (45)	Call back group 193	Female: 133<br>Male: 57<br>Trans/non-binary: 3	16: 45<br>17: 148	White: 81<br>Caribbean: 45<br>African: 26<br>Asian: 14<br>Other ethnicity: 27	Heterosexual - 157<br>Homosexual – 15<br>Bisexual - 21	Unstated
		Non-call back group 261	Female: 179<br>Males: 90<br>Trans/non-binary: 1	16: 74<br>17: 187	White: 87<br>Caribbean: 90<br>African: 27<br>Asian: 15<br>Other ethnicity: 42	Heterosexual - 227<br>Homosexual – 9<br>Bisexual - 25	Unstated

	Day et al. 2021 (46)	34 confirmed new HIV results from 144 people with reactive HIV results.	Female: 2<br>Male: 32	Median 28<br>Range 21-50	White British or White Other: 20<br>Black, Asian and Minority Ethnic groups: 4<br>Other: 10	Gay and bisexual MSM – 30<br>Heterosexual men – 2<br>Heterosexual women - 2	Unstated
**SH:24**	Barnard et al. 2018 (32)	5747 (3198 clinic, 2549 online)	Female: 3258<br>Male: 2489	16-20yrs – 358<br>20-25yrs – 1516<br>25-30yrs – 1798<br>30-35yrs – 895<br>35+yrs - 1180	White British – 2233<br>White other – 829<br>Black African – 560<br>Black Caribbean – 480<br>Black other – 496<br>Mixed white black African or Caribbean' – 225<br>South Asian – 90<br>Other – 600<br>Unstated - 229	Heterosexual – 4731<br>Homosexual – 651<br>Bisexual – 165<br>Unstated - 200	IMD quintile<br>1 (most deprived) – 2115<br>2 – 2394<br>3 – 974<br>4 – 219<br>5 (least deprived) – 29
	Barnard 2020 (Chapter 6) (47)	20	Female: 7<br>Male: 12<br>Trans: 1	16-19yrs – 1<br>20-24yrs – 10<br>25-30yrs - 9	White – 8<br>Mixed – 3<br>Asian – 2<br>Black – 6<br>Other - 1	Heterosexual – 13<br>Homosexual – 5<br>Bisexual - 2	Unstated
	Syred et al. 2019 (48)	Pre-intervention - 6253	Female: 4030<br>Male: 2223	16-24yrs – 3351	Black and Minority Ethnic – 642	Men who have sex with Men (MSM) – 367	IMD quintile<br>1 (most deprived) – 580<br>2 – 1364<br>3 – 1399<br>4 – 1614<br>5 (least deprived) - 1296
		Post- intervention - 7772	Female: 4968<br>Male: 2804	16-24yrs – 4120	Black and Minority Ethnic – 878	MSM – 530	IMD quintile<br>1 (most deprived) – 794<br>2 – 1705<br>3 – 1847<br>4 – 1886<br>5 (least deprived) - 1538

	Turner et al. 2018 (20)	Camberwell Sexual Health Clinic – 4172	Female: 1340<br>Male: 921	16-19yrs – 201<br>20-24yrs – 472<br>25-29yrs – 508<br>30-34yrs – 365<br>35+yrs – 715	White – 825<br>Mixed – 178<br>Asian – 59<br>Black or Black British – 939<br>Other – 208<br>Unstated - 52	MSM – 251	Unstated
		Spontaneous SH:24 – 5632	Female: 2746<br>Male: 1516	16-19yrs – 194<br>20-24yrs – 1282<br>25-29yrs – 1605<br>30-34yrs – 650<br>35+yrs – 531	White – 2850<br>Mixed – 353<br>Asian – 107<br>Black or Black British – 768<br>Other – 105<br>Unstated - 79	MSM – 505	Unstated
		Triage – 1266	Female: 416<br>Male: 474	16-19yrs – 55<br>20-24yrs – 205<br>25-29yrs – 262<br>30-34yrs – 146<br>35+yrs – 222	White – 461<br>Mixed – 93<br>Asian – 21<br>Black or Black British – 270<br>Other – 32<br>Unstated - 13	MSM – 94	Unstated
	Turner et al. 2019 (33)	Time period 1 – 43491 clinic visits<br>Time period 2 – 51191 clinic visits, 11768 online orders	Unstated	Unstated	Unstated	Unstated	Unstated
	Wilson et al. 2017 (49)	Intervention group - 1031	Female: 604<br>Male: 424<br>Trans: 3	16-19yrs – 206<br>20-24yrs – 440<br>25-30yrs – 385	White – 779<br>Black/African/Caribbean/black British – 81<br>Asian/Asian British – 70<br>Mixed – 89<br>Other – 12	MSM – 129<br>Other – 890<br>Unstated – 12	Unstated
		Control group - 1032	Female: 609<br>Male: 422<br>Trans: 1	16-19yrs – 220<br>20-24yrs – 432<br>25-30yrs – 380	White – 749<br>Black/African/Caribbean/black British – 110	MSM – 133<br>Other – 888<br>Unstated – 11	Unstated

					Asian/Asian British – 57<br>Mixed – 99<br>Other – 17		
	Wilson et al. 2019 (50)	Intervention group - 244	Female: 130<br>Male: 113<br>Trans: 1	16-19yrs – 96<br>20-24yrs – 96<br>25-30yrs – 52	White/WB – 176<br>Black/black British – 18<br>Asian/Asian British – 33<br>Mixed – 14<br>Other – 3	MSM – 14<br>Other – 224<br>Unstated – 6	Unstated
		Control group - 284	Female: 142<br>Male: 141<br>Trans: 1	16-19yrs – 118<br>20-24yrs – 110<br>25-30yrs – 56	White/WB – 176<br>Black/black British – 25<br>Asian/Asian British – 34<br>Mixed – 26<br>Other – 5	MSM – 21<br>Other – 258<br>Unstated – 5	Unstated
**TakeATestUK.com**	Page et al. 2021 (51)	Mini tube - 1515	Female:1051<br>Male:460<br>Trans:0<br>Other:4	Mean (95% CI): 27.4 (27.1-27.8)<br>Median (IQR): 26 (22-31)	Any other Asian – 2<br>Any other Black – 6<br>Any other mixed – 11<br>Any other white – 41<br>Bangladeshi – 6<br>Black African – 25<br>Black Caribbean – 81<br>Chinese – 1<br>Indian – 5<br>Pakistani – 13<br>Unknown/not specified – 26<br>White and Asian – 17<br>White and Black African – 2<br>White and Black Caribbean – 79<br>White British – 1147<br>White Irish - 7	Heterosexual man – 353<br>Heterosexual woman – 977<br>Bisexual man – 20<br>Bisexual woman – 18<br>MSM exclusive – 87<br>Women who have sex with women (WSW) exclusive – 56<br>Heterosexual trans woman - 0<br>Unknown/not specified – 4	Unstated
		Dried blood spot - 4155	Female: 2788<br>Male: 1357<br>Trans: 2	Mean (95% CI): 27.3 (27.1-27.5)<br>Median (IQR): 26	Any other Asian – 2<br>Any other Black – 6<br>Any other mixed – 11	Heterosexual man – 1012<br>Heterosexual woman – 2617	Unstated

			Other: 8	(2-31)	Any other white – 41<br>Bangladeshi – 6<br>Black African – 25<br>Black Caribbean – 81<br>Chinese – 1<br>Indian – 5<br>Pakistani – 13<br>Unknown/not specified – 26<br>White and Asian – 17<br>White and Black African – 2<br>White and Black Caribbean – 79<br>White British – 1147<br>White Irish - 7	Bisexual man – 67<br>Bisexual woman – 95<br>MSM exclusive 278<br>WSW exclusive – 76<br>Heterosexual trans woman - 2<br>Unknown/not specified – 8	
**Umbrella**	Banerjee et al. 2018 (35)	Patients who requested home-based kits – 9258	Female: 5986<br>Male: 3258<br>Trans: 14	16-24yrs – 6033<br>25+yrs – 3225	White – 6648<br>Black/black British – 892<br>Asian/Asian British – 558<br>Other – 920<br>Unstated – 240	Heterosexual male – 2606<br>Heterosexual female – 5986<br>MSM – 652<br>Trans – 14	Unstated
		Patients who returned home-based kits - 4475	Femae: 3104<br>Male: 1367<br>Trans: 4	16-24yrs – 2868<br>25+yrs – 1607	White – 3375<br>Black/black British – 351<br>Asian/Asian British – 184<br>Other – 394<br>Unstated – 171	Heterosexual male – 1039<br>Heterosexual female – 3104<br>MSM – 328<br>Trans – 4	Unstated
		Patients tested in clinic - 19193	Female: 10861<br>Male: 8306<br>Trans: 26	16-24yrs – 9654<br>25+ yrs – 9539	White – 7996<br>Black/black British – 4026<br>Asian/Asian British – 2167<br>Other – 2160<br>Unstated – 2844	Heterosexual male – 6602<br>Heterosexual female – 10662<br>MSM – 1675<br>Trans – 24<br>Bisexual – 199<br>WSW – 31	Unstated
	Banerjee et al. 2020	Patients who requested home-	Female: 10686	16-24yrs – 8819<br>≥25yrs – 7792	White – 11,519<br>Black – 1692	Heterosexual (M) – 5746<br>Heterosexual (F) – 10,667	Unstated

	(36)	based testing kit – 16,611	Male: 5889<br>Trans: 36		Asian – 1148<br>Other – 1770<br>Not stated - 482	Trans – 36<br>MSM – 138<br>Bisexual – 18<br>WSW - 6	
		Patients who returned home-based testing kits with sufficient quality of blood for testing – 9033	Female: 6004<br>Male: 3018<br>Trans: 11	16-24yrs – 4623<br>≥25 years - 4419	White – 6588<br>Black – 803<br>Asian – 473<br>Other – 971<br>Not stated - 198	Heterosexual (M) – 2875<br>Heterosexual (F) – 5985<br>Heterosexual trans – 11<br>MSM – 138<br>Bisexual – 18<br>WSW - 6	Unstated
		Patients who had serological testing in clinic	Female: 8236<br>Male: 8422<br>Trans: 37	16-24yrs – 6616<br>≥ 25 - 10079	White – 6331<br>Black – 3296<br>Asian – 1910<br>Other – 1796<br>Not stated - 3362	Heterosexual (M) – 6617<br>Heterosexual (F) – 8093<br>Heterosexual trans – 37<br>MSM – 1788<br>Bisexual – 95<br>WSW - 65	Unstated
	Manavi et al. 2017 (52)	Test kits requested – 5310<br>(number of participants unstated)	Female kits: 3513<br>Male kits: 1787<br>Trans kits: 10	Unstated	Unstated	Unstated	IMD rank<br><5000 – 1855<br>5000-14999 – 2095<br>15000+ - 1321

<u>Table Key:</u>

IMD: Index of Multiple Deprivation Unstated: unstated/refused/missing data SD: standard deviation
MSM: men who have sex with men WSW: women who have sex with women SRH: Sexual and Reproductive Health

**Bracebridge et al. 2012 have labelled IMD quintile 1 as least deprived and IMD quintile 5 as most deprived in their paper. This may be an error and makes this data difficult to interpret. The corresponding author has been contacted for clarification.*

Supplemental File 4: Study Summaries and Key Findings

Service Title	Authors (date)	Study type	Study aims	Description of study design	Inclusion/ exclusion criteria	Key results
**eSexual Health Clinic (eSHC)**	Aicken et al. 2018 (37)	Qualitative interviews	To understand use and experience of the eSHC to inform future evaluation and refinement	• Telephone interviews with patients who had used the eSHC<br>• Framework analysis carried out with codes developed from the sSHC	**Excluded:** symptomatic or coinfected patients, aged <16yrs, unable to read English, no phone number provided	• Key themes included rapidity, protecting privacy, choice and seeking peace of mind<br>• Subthemes included technology constraints, concerns with accessing results in public, simple and discreet treatment collection being positive and the trustworthiness conferred by the pathway being integrated within the NHS
	Estcourt et al. 2017 (38)	Non randomised, exploratory proof of concept study	To assess the safety and feasibility of eSHC	• Chlamydia positive patients contacted 2 weeks after receiving their diagnosis<br>• Outcomes included appropriate care management, time to treatment and proportion of partners treated<br>• Chlamydia-negative users sent a brief acceptability survey	**Excluded:** symptomatic or coinfected patients, already receiving treatment for undiagnosed chlamydia, <16yrs, unable to read English	• ~75% of users accessed the pathway of whom 60% managed solely online<br>• ~25% users contacted the helpline<br>• Most patients collected treatment from their allocated pharmacy<br>• The day after receiving diagnosis 76% of GUM patients and 67% of NCSP Checkurself patients had collected treatment<br>• 29% of GUM patients and 24% of NCSP Checkurself patients accessed online health information

	Gibbs et al. 2018 (39)	Mixed methods evaluation	To evaluate the eSHC	• Data collected on eSHC webpage interactions and a survey to establish acceptability for chlamydia positive patients<br>• Qualitative interviews with 36 users (see Aicken et al. 2018)<br>• Survey of chlamydia-negative users<br>• Acceptability analysed descriptively	See Estcourt et al. 2017 and Aicken et al. 2018	• 82-92% of patients accessed results within 5 days. Of these, 97% accessed their results on the day they received the text<br>• For key findings from qualitative interviews, see Aicken et al. 2018<br>• 91% of chlamydia positive patients surveyed were pleased with the service, 66% of previous testers preferred the eSHC, 99% felt they received sufficient health information<br>• Chlamydia negative users: 98% of first-time testers were pleased with the service, 90% of previous testers would use the eSHC again - 53% of those preferred the eSHC to alternative services
**Freetest. me**	Dolan et al. 2014 (40)	Randomised experiment	To explore the effect on chlamydia test return rates of non-cash financial incentives, and the influence of socioeconomic status	• Test requests randomly allocated to intervention (incentive) or control (no incentive)<br>• Test kits sent identifiable bar codes for incentive<br>• Logistic regression was conducted using postcodes to identify socioeconomic status	**Included:** individuals using site during the study period	• Those requesting kits mostly female, white, displayed higher risk behaviours (e.g. 2+ partners in the last 12 months), less deprived, over 40% had completed a chlamydia test/over 30% had tested positive in the last year<br>• Return rate was 71%, only small differences between incentives, none statistically significant<br>• Individuals aged 15-19 and of lower socioeconomic position less likely to return tests

**Letstalkabout it**	Gasmelsid et al. 2021 (34)	Retrospective service evaluation	To determine whether online screening is accessible by those patients most at need by comparing the demographics and number of asymptomatic chlamydial infections detected online and in clinic.	• Single service data<br>• Comparison of the demographic characteristics and number of asymptomatic chlamydial infections detected via an online postal self-sampling service and in clinic<br>• Two time periods: Time 1- pre-introduction of online postal self-sampling ((Sept 2014-March 2015); Time 2 and post-introduction of the online service (Sept 2017-March 2018)	**Included:** People testing positive for chlamydia in Solent NHS Trust services (clinic-based and online) between September 2017-March 2018	• The demographic characteristics of individuals accessing services was similar in clinic and online services, and remained stable between Time 1 and Time 2.<br>• The majority of patients diagnosed were <25 years old, of white ethnicity, heterosexual and women.<br>• There were no differences in IMD before those diagnosed in Time 1 and Time 2<br>• There was a significantly higher proportion of service users who identified as gay, bisexual or other men who have sex with men in Time 2 compared to Time 1<br>• There was a significantly higher proportion of service users of Black, Asian and Minority ethnicity in Time 2 compared to Time 1<br>• Patients diagnosed in clinic were significantly more likely to wait more than a week for treatment than those diagnosed through online services.
**NCSP**	Woodhall et al. 2012(5)	Quantitative retrospective data analysis; website evaluation	To describe and evaluate access to the NCSP's online chlamydia testing service	• Chlamydia testing data analysed to describe trends and proportion of internet tests, and describe online testing by area<br>• Descriptive comparative analyses conducted of online vs face-to-face users<br>• 90 websites evaluated on health promotion and clinical signposting information	**Included:** had test codes for internet testing, aged 16-24	• 5.3% of tests ordered online 2006-2010 - <0.5% in 2006 to a maximum of 7.1% in 2009 (varied by area, some <1% and others <40%)<br>• Online users more likely men, aged 20-24, of white ethnicity and less deprived<br>• Women more likely to have had a new sexual partner in the 3 months before testing, or +1 in the previous year, than face-to-face users<br>• A high proportion of online tests resulted in positive chlamydia diagnoses<br>• Internet testers spread out in levels of deprivation, whereas face-to-face testers were more deprived

**North East Essex PCT**	Bracebridge et al. 2012 (41)	Cross-sectional study	To quantify uptake and test-positivity rates, identify factors associated with screening and compare costs of the intervention with the NCSP	• Associations examined between personal characteristics and study outcomes: test uptake, service registration and test positivity<br>• Demographic data obtained through test registration	**Included:** aged 18-24, residing within the boundaries of NE Essex PCT	• 82% requested online screening<br>• Screening uptake less likely among men, less deprived and over 20's<br>• Having 2+ partners in the previous year strongly associated to a positive diagnosis<br>• 95.4% of chlamydia positive individuals and all notified partners requested postal treatment<br>• Costs per screening test and positive diagnosis were 1.66 and 3.5 times more than the NCSP
**Saving lives**	Page et al. 2019 (42)	Observational study	To ascertain how DBS HIV kits compared with MT kits in this postal testing service.	• Single service dataset analysed to compare online requested MT and DBS HIV test kits<br>• Analyses evaluated the online request, return and results of the two different bloods sampling techniques that were used sequentially by the service	**Included:** All service users who ordered an HIV test kit during the study time period, and had consented to their anonymised data to be shared by a third- party organisation.	• Similar demographic characteristics of those accessing MT compared to those accessing DBS: 63% women, 90% white British, 86% heterosexual, median age 26)<br>• No difference in return rates between MT and DBS. However statistically significant higher proportion of successful sample processing with DBS (98.8%) compared with MT (55.7%).<br>• Higher proportion of false positives with MT (5.4%) *c.f.* DBS (0.0%)

**Sexual Health London**	Day et al. 2020 (43)	Retrospective service evaluation	To report the rate of recent sexual assault (SA) disclosure amongst users of SHL, and identify the outcomes of their call back discussions.	• Single service dataset<br>• Service users reporting that they have been a victim of a recent sexual assault are contacted by the health advisor team<br>• Outcomes included successful phone contact with patient, referral to Sexual Assault Referral Centre, Intervention by SHL team, test kit return, diagnosis of STI	**Included:** All patients using SHL between 01/01/2020 and 18/02/2020 who triggered a call back for sexual assault	• 0.5% (242/45841) users triggered at least one call back for a SA<br>• Majority of users were female (54.0%), heterosexual (72%), of white ethnicity (44.4%) and 80.4% had attended a sexual health clinic previously.<br>• 79.3% (192/242) of call backs were successfully.<br>• Of those that were contactable, 45% (87/192) of confirmed a recent SA and 52.6% (101/192) stated that they had made an error on the triage. 76.2% (77/101) of the latter were male.<br>• 92.6% (224/242) kits were dispatched, and of these 73.7% (165/224) kits were returned and tested during the study period<br>• For 90% (78/87) of those reporting a SA, no onward referral was made.
	Day et al. 2021 (44)	Retrospective service evaluation	To assess the sexual health needs, sexual practices, STI/HIV positivity and satisfaction rates of trans and non-binary users of Sexual Health London	• Single service dataset<br>• Demographic characteristics and outcomes of service users identifying as transgender, non-binary/gender fluid or 'other' (TNB) registering to use SHL.<br>• Outcomes included: sexual practices, sexual/reproductive healthcare needs and prior SHC attendance, service outcomes, STI test results and satisfaction scores.	**Included:** All people identifying as TNB when registering for SHL between 20th April 2019 and 31st December 2019	• 0.42% (540/119329) of registrants identified as transgender, non-binary/gender fluid or 'other'.<br>• 463 kits were placed, and 355 kits were returned from 302 unique users. No difference in kit return rate compared to cisgender individuals<br>• The odds of being of Black, Asian or Minority Ethnicity were 1.2 times higher compared with cisgender individuals.<br>• The odds of returning a blood sample were 1.6 times higher compared with cisgender individuals<br>• TNB service users were significantly more likely to engage in sex work, and reported similar rates of chemsex, group sex and fisting to that seen with MSM.<br>• 95% (50/51) of users would recommend SHL to friends/family<br>• 85.4% (70/82) gave a 5/5 star service rating

	Day et al. 2020 (45)	Retrospective service evaluation	To report the safeguarding concerns and outcomes of 16-17year olds accessing SHL	• Single service data<br>• SHL uses questions adapted from the Spotting the Signs proforma<br>• Demographic characteristics and outcomes of those 16-17 year olds whose responses to the questions triggered a phone call ((‘call back’ (CB)) from a health advisor, compared with those that didn’t<br>• Outcomes included: type & number of safeguarding triggers, CB outcomes, safeguarding outcomes among CB cases, STI test kits ordered and returned, STI test results.	**Included:** All 16 and 17 year olds who triggered a CB from a health advisor	• 42.5% (193/454) service users triggered one CB, and 7 triggered 2 when ordering a second kit (i.e. they were 200 CBs triggered)<br>• The most common reasons for triggering a call back were related to drug and alcohol use (27%0. Partner’s age imbalance (18% and involvement with social or mental health services (8%_<br>• All users received at least one CB attempt, and 84.5% had a successful call back.<br>• 37.9% had a trigger downgraded (mainly because they had misread, misinterpreted or teicked the question in error. 6.5% disclosed additional or more serious concerns<br>• 35.5% were referred to or attend a sexual health clinic<br>• 8.5% were referred to the child protection team, mostly because they were not contactable or became uncontactable.<br>• 7% of cases involved a discussion/referral to social services.
	Day et al. 2021 (46)	Retrospective service evaluation	To identify the characteristics and transfer to care rates of those who have a reactive HIV test result via SHL	• Single service data<br>• Demographics and outcomes of those users with a reactive HIV test result	**Included:** People with reactive HIV test results between 8th January 2018-31st December 2019	• 0.097% (144/148,257) had a reactive HIV result<br>• 20.8% (30/144) were known to be living with HIV<br>• 29.8% (34/114) were confirmed HIV positive<br>• 59.6% (68/114) were found to be HIV negative (i.e. were false positives)<br>• In 10.5% (12/114) the result was unknown<br>• Of those confirmed as new HIV diagnoses, all service users transitioned to a HIV outpatient clinic. The majority were male (94.1%), men who have sex with men (88.2%), and of white ethnicity (58.8%).

**SH:24**	Barnard et al. 2018 (32)	Cross-sectional study	To compare the characteristics of e-STI service users with clinic users, and OPSS kit returners with non-returners	• Service activity data collected from sexual health clinics in Lambeth and Southwark<br>• Complete case analysis carried out using logistic regression	**Included:** residents of Lambeth and Southwark<br><br>**Excluded:** activity codes outside the remit, testing by prisoners	• Online services most popular with users aged 20-30 years, women, white British ethnicity, homosexual or bisexual individuals, those who receive negative results, and are less deprived<br>• Women who were 'mixed white black African or Caribbean' had lower odds of using online services compared to men in this group<br>• Homosexual women were more likely to use online services than homosexual men<br>• The most likely groups to return samples were >20 years ($p<0.05$) and white British
	Barnard 2020 (Chapter 6) (47)	Qualitative interviews	To describe the experiences, barriers and facilitators of SH:24 in Lambeth and Southwark	• Stratified purposive sampling of consenting trial participants (Wilson et al. 2017)<br>• Thematic analysis	See Wilson et al. 2017	• Key themes were trust, subjective norms, privacy, self-efficacy, convenience and perceived risk of infection<br>• Subthemes included service reliability and confidentiality, comfort and control, concealing testing, improved trust and subjective norms over time, risk of infection, self-sampling and validity of results

	Syred et al. 2019 (48)	Observational study	To describe user choice of OPSS orders and diagnoses in a 'choose to test' intervention	• An online 'choose to test' intervention was piloted in Essex, UK<br>• Users given a personalized test package<br>• Users could add or remove tests<br>• Outcomes included test package edits, cost of tests and diagnoses	n/a	• Slight increase in MSM and more deprived groups occurred after the intervention<br>• 17.2% of MSM removed tests, 67.3% of BME users added a syphilis test, 59.8% of users in neither group added HIV and syphilis tests<br>• Orders from women and BME groups most likely to be modified, orders from 16-24s, MSM and symptomatic users least likely to be modified<br>• Number of positive chlamydia or gonorrhoea diagnoses did not significantly change<br>• Where users are given 'choice to test', most will choose chlamydia, gonorrhoea, HIV and syphilis<br>• Costs are saved through a reduction in HIV and syphilis tests ordered
	Turner et al. 2018 (20)	Observational study	To investigate the effect of decision-making on resource allocation in a clinic after the introduction of an e-STI service in Lambeth and Southwark	• Demographic data, type of STI test and area of residence were collected before and after the intervention<br>• Outcomes analysed included testing volume and complexity in clinic, and test positivity between pathways	**Included:** residents of Lambeth and Southwark only<br>Excluded: codes lacking from clinic visit, prisoners, <16yrs or >100yrs	• Online testing increased the volume of testing, clinic visits were proportionally more complex<br>• The greatest proportion of 16-19s tested in clinic<br>• Women were most likely to use the online service but less likely to return tests, MSM were comparatively likely to use clinic or e-STI services<br>• Most online or triaged users were asymptomatic - reflective of the encouragement to use clinics if displaying symptoms<br>• More tests returned by spontaneous online users than triage patients (p=0.01)

	Turner et al. 2019 (33)	Case study analysis and model generation	To establish cost-effectiveness of an OPSS service, and explore cost per diagnosis in different scenarios	• Records of demographics, sexual orientation and clinical information from clinic visitations were captured<br>• Online service data collected<br>• Case study analysis conducted on cost-effectiveness of testing prior and post e-STI service	**Excluded:** codes lacking from clinic visit, prisoners, <16yrs or >100yrs	• Clinic testing rates remained stable after SH:24 was introduced, but online testing increased overall testing volume by 27% from 2014-16<br>• 37% of tests were ordered online by 2016<br>• Average test positivity rates higher in clinic than online<br>• Average monthly diagnoses and annual cost of testing increased from 2014-16, but cost per test and per diagnosis decreased<br>• Return rates impact cost if they are below 60%; this study observed an over 75% return rate
	Wilson et al. 2017 (49)	Single-blind randomised control trial (RCT)	To assess the effectiveness of an OPSS service compared with face-to-face services	• Participants recruited from community settings, online and through clinics<br>• Participants randomly allocated to intervention (text message with e-STI service link) or control (text message with local clinic information)<br>• Staff were blinded to the allocation<br>• Outcomes included test completion, time to testing or treatment, positive tests and acceptability	**Included:** aged 16-30, resident in Lambeth or Southwark, 1+ sexual partner in the 12 months prior, willing to complete a test, internet access<br><br>**Excluded:** unable to read English or provide consent	• STI testing at 6 weeks higher in the intervention group (50% vs 26.6%, p<0.001) with no evidence of heterogeneity across population groups so could be targeted to higher-risk groups<br>• Time to test shorter in the intervention group (28.8 days vs 36.5 days, p<0.001)<br>• Proportion of diagnoses or individuals treated, and time to treatment were not statistically significant (patients required to get treatment in clinic)<br>• 71% surveyed found the intervention acceptable

	Wilson et al. 2019 (50)	Secondary data analysis of an e-STI RCT in Lambeth and Southwark	To examine the effect of an e-STI service on testing uptake on people who had never previously tested (never-testers)	• Data analysed on never-testers<br>• Outcomes included testing at 6 weeks, time to test, positive results, test completion by service, and acceptability<br>• Interactions examined between demographics	See Wilson et al. 2017	• Data available for 87% of the intervention and 79% of the control group<br>• Return of STI test at 6 weeks was higher for the intervention group (~45% vs ~25%, p<0.001)<br>• Intervention reduced time to test at 42 days<br>• Face-to-face community recruitment increased the effectiveness of the intervention<br>• 74.6% surveyed found the intervention acceptable<br>• There were greater proportions of men, 16-19 year old's, and Asian or Asian British ethnicities in the never-testers<br>• Further barriers - around 54% did not test

**TakeATestUK.com**	Page et al. 2021 (51)	Observational study	To ascertain how DBS HIV and syphilis kits compared with MT kits in this postal testing service	• Single service dataset analysed to compare online requested MT and DBS HIV test kits<br>• Analyses evaluated the online request, return and results of the two different bloods sampling techniques that were used sequentially by the service.<br>• Secondary objectives were to describe the number of kit requests to obtain one successfully processed result, and the proportion of false-positive results for DBS and MT.	**Included:** All service users who ordered an HIV/syphilis test kit during the study time period, and had consented to their anonymised data to be shared by a third- party organisation.	• Similar demographic characteristics of those accessing MT compared to those accessing DBS (majority female (69.4% vs 67.1), white British (75.7% vs 73.2%) heterosexual (87.8% vs 87.3%), median age 26 (IQR 22-31)<br>• No difference in proportion of people who returned STI kit who also returned HIV/STS sample (88.2% for MT, 87% for DBS, p= 0.340).<br>• Statistically significant higher proportion of successful sample processing with DBS (94.6%) compared with MT (54.4%), p=<0.001.<br>• Higher proportion of MT samples were reactive for HIV compared with DBS (6.2% vs 1.1%, p=<0.001). However, higher proportion of false positives with MT than DBS (5.2% of all successfully processed MT samples *c.f.* 0.4% of DBS)<br>• Lower proportion of STS samples were reactive (1.1% for MT samples *c.f.*0.7% of DBS), with 0.4% of successfully processed MT samples being false positive *c.f.* 0.0% for DBS.)
**Umbrella**	Banerjee et al. 2018 (35)	Retrospective service evaluation	To evaluate the rates of uptake and return of OPSS kits and compare patient demographics and clinical outcomes in home and clinic testers	• Retrospective data compared between home test users and clinic test users<br>• Outcomes included time before kit return, time to treatment, positivity and treatment rates	**Included:** patients who took home or clinic based STI tests in the study period, >16yrs, residing in Birmingham/Solihull	• A third of patients requested home tests, 48% returned kits<br>• Home sampling was more popular among those aged 16-24, of white ethnicity, heterosexual female and asymptomatic (p<0.001)<br>• Home sampling was less popular in black/black British and Asian/British Asian groups (p<0.001)<br>• Positivity rates higher in clinic<br>• Treatment rates lower in the home self-sampling group and time-to-treat longer than in clinic groups

	Banerjee et al. 2020 (36)	Retrospective service evaluation	To evaluate the uptake, return rate and new diagnosis rates of home-base testing in comparison with clinic-based for HIV, STS, and hepatitis B	• Retrospective data compared between home test users and clinic test users<br>• Outcomes included total number of patients in the two groups according to patient demographics, and presence of symptoms, number of patients returning home kit with sufficient quantity of blood compared to serological testing in clinci, overall retunr rate of home-based testing kits, number of patients with reactive results using home-based kits compared to clinic, outcomes of patients with reactive/equivcal tests in both groups.	**Included:** Patients using home-based testing kits or attending clinics in the Birmingham & Solihull area between July-December 2017	• Home sampling was more popular amongs those aged 16-24, white, female patients (p<0.001.<br>• Home sampling was less popular in Black and Asian groups (p<0.001)<br>• Only 54% (9033/16,611) of home-based test kits were returned with sufficient quantity of blood for testing.<br>• False positivity rate was significantly higher for HIV and Hepatitis B in the home based group.<br>• 26/75 of HIV, 71/146 of STS, and 12/15 Hep B reactive results from home-based sampling were unconfirmed.<br>• Only 1/75 of reactive HIV tests, 8/146 for STS, and 0/15 for Hepatitis B from home-based test kits were true-positives, new cases.
	Manavi et al. 2017 (52)	Observational study	To establish which factors influence return of OPSS kits	• Retrospective data collected including demographic information, sexual history and symptoms<br>• This data linked to the laboratory system to confirm which individuals returned requested kits	**Included:** use of the service within the study period	• 58.4% of kits were returned (61.2% of women vs 53.1% of men, p<0.001, and 10% of transgender individuals). MSM had similar rates of kit return to women; heterosexual men were less likely to return kits<br>• Those who returned tests were less deprived<br>• Patients requesting home kits rather than pharmacy-collection were more likely to return them<br>• Symptomatic patients less likely to return kits, potentially due to clinic attendance

Supplemental File 5 – Mixed Methods Appraisal Tool (MMAT)

Author (date)	S1: Are there clear research questions?	S2: Do the collected data allow to address the research questions?	1.1. Is the qualitative approach appropriate to answer the research question?	1.2. Are the qualitative data collection methods adequate to address the research question?	1.3. Are the findings adequately derived from the data?	1.4. Is the interpretation of results sufficiently substantiated by data?	1.5. Is there coherence between qualitative data sources, collection, analysis and interpretation?	Comments
Aicken et al. 2018 (37)	YES	YES	1.1 YES	1.2 YES	1.3 YES	1.4 YES	1.5 YES	
Barnard 2020 (Chapter 6) (47)	YES	YES	1.1 YES	1.2 YES	1.3 YES	1.4 YES	1.5 YES	
			**2.1. Is randomization appropriately performed?**	**2.2. Are the groups comparable at baseline?**	**2.3. Are there complete outcome data?**	**2.4. Are outcome assessors blinded to the intervention provided?**	**2.5 Did the participants adhere to the assigned intervention?**	
Dolan 2014 (40)	YES	YES	2.1 YES	2.2 YES	2.3 YES	2.4 NO	2.5 YES	
Wilson 2017 (49)	YES	YES	2.1 YES	2.2 YES	2.3 YES	2.4 YES	2.5 YES	
Wilson 2019 (50)	YES	YES	2.1 YES	2.2 YES	2.3 YES	2.4 YES	2.5 YES	This was secondary analysis of RCT data. The MMAT has been completed

								based on the criteria of the original RCT (Wilson et al. 2017)
			**3.1. Are the participants representative of the target population?**	**3.2. Are measurements appropriate regarding both the outcome and intervention (or exposure)?**	**3.3. Are there complete outcome data?**	**3.4. Are the confounders accounted for in the design and analysis?**	**3.5. During the study period, is the intervention administered (or exposure occurred) as intended?**	
Banerjee 2018 (35)	YES	YES	3.1 YES	3.2 YES	3.3 YES	3.4 NO	3.5 YES	
Banerjee 2020 (36)	YES	YES	3.1 YES	3.2 YES	3.3 YES	3.4 NO	3.5 YES	
Barnard 2018 (32)	YES	YES	3.1 YES	3.2 YES	3.3 YES	3.4 YES	3.5 YES	
Bracebridge 2012 (41)	YES	YES	3.1 YES	3.2 YES	3.3 YES	3.4 YES	3.5 YES	
Day 2020 (43)	YES	YES	3.1 YES	3.2. YES	3.3. NO	3.4 NO	3.5. YES	
Day 2021 (44)	YES	YES	3.1 CAN'T TELL	3.2. YES	3.3. NO	3.4. NO	3.5 YES	
Day 2020 (45)	YES	YES	3.1. YES	3.2. YES	3.3. NO	3.4 NO	3.5 YES	
Day 2021 (46)	YES	YES	3.1. YES	3.2 YES	3.3. NO	3.4 NO	3.5 YES	
Estcourt 2017 (38)	YES	YES	3.1 YES	3.2 YES	3.3 YES	3.4 NO	3.5 YES	
Gasmelsid 2021 (34)	YES	NO	3.1 NO	3.2. NO	3.3. NO	3.4 NO	3.5 YES	

Page 2019 (42)	YES	NO	3.1 YES	3.2 YES	3.3. YES	3.4 NO	3.5 YES	
Page 2021 (51)	YES	NO	3.1 YES	3.2 YES	3.3. YES	3.4 NO	3.5 YES	
Syred 2019 (48)	YES	YES	3.1 YES	3.2 YES	3.3 YES	3.4 YES	3.5 YES	
Turner 2018 (20)	YES	YES	3.1 YES	3.2 YES	3.3 YES	3.4 YES	3.5 YES	
Turner 2019 (33)	YES	YES	3.1 YES	3.2 YES	3.3 YES	3.4 NO	3.5 YES	
			**4.1. Is the sampling strategy relevant to address the research question?**	**4.2. Is the sample representative of the target population?**	**4.3. Are the measurements appropriate?**	**4.4. Is the risk of nonresponse bias low?**	**4.5. Is the statistical analysis appropriate to answer the research question?**	
Manavi 2017 (52)	YES	YES	4.1 YES	4.2 YES	4.3 YES	4.4 YES	4.5 YES	
Woodhall 2012 (5)	YES	YES	4.1 YES	4.2 YES	4.3 YES	4.4 YES	4.5 YES	
			**5.1. Is there an adequate rationale for using a mixed methods design to address the research question?**	**5.2. Are the different components of the study effectively integrated to answer the research question?**	**5.3. Are the outputs of the integration of qualitative and quantitative components adequately interpreted?**	**5.4. Are divergences and inconsistencies between quantitative and qualitative results adequately addressed?**	**5.5. Do the different components of the study adhere to the quality criteria of each tradition of the methods involved?**	
Gibbs 2018 (39)	YES	YES	5.1 YES	5.2 YES	5.3 YES	5.4 YES	5.5 YES	

Supplemental File 6: Summaries of the ten online postal STI services

Service	Setting	Summary of service or intervention
**eSHC** (37–39)	Greater London	• Individuals undergo STI testing at one of two included genitourinary (GUM) clinics or via an online postal self-sampling service provided by six NCSP areas in South London<br>• All eligible patients receive an discreetly worded text message from the secure NHS SMS system stating that their results are available and can be viewed using an attached link for a password-protected online application<br>• Patients log on with their date of birth and either their clinic or phone number<br>• If the result is positive, patients are offered and may provide consent for their use of a remote, self-directed online chlamydia pathway. They complete an online consultation to provide routinely collected clinical and public health surveillance data, and they are provided with trusted links to access information about their condition. Patients can then nominate one of 30 participating pharmacies from which they can collect their treatment<br>• If users receive a negative result, health promotion material is provided<br>• If users opt out of the online care pathway, they redirected to traditional face-to-face care<br>• A helpline staffed by research health advisors is accessible from 9:00am-5:00pm on weekdays<br>• If users describe factors such as allergies, symptoms or drug use during their online consultation then they are directed to call the helpline whose staff will facilitate their access directly to face-to-face care<br>• Treatment for sexual partners is recommended; sexual partners can be notified and linked to the record of their sexual partner
**Freetest.me** (40)	All areas of England except within the North East Strategic	• Preventx Limited provide an online and text service called Freetest.me<br>• Individuals request a postal kit online; samples are returned by post using the prepaid return box<br>• Individuals choose to be informed that their results are available for access by either text or email. They can opt in to being telephoned if results are positive

	Health Authority	• Results can be viewed online via an online tracking system<br>• A reminder text is sent if samples have not been returned within 18 days, and samples not returned within 30 days of the request date are deemed invalid
**Letstalkaboutit** (34)	Hampshire	• OPSS service offered to residents of Hampshire, through Solent NHS Trust.<br>• Introduced county-wide in 2015.<br>• Local sexual health clinics sign post eligible service users to the Letstalkaboutit website. Service users request a kit online, the kit is posted to their home address, and they post the samples back to the laboratory.<br>• Results via text or phone.
**NCSP** (5)	England	• The English National Chlamydia Screening Programme provides no-cost opportunistic testing for young people and easy access to treatment<br>• Sexually active patients attending healthcare settings such as a general practice (GP), local pharmacies, and sexual health, abortion or reproductive health services are offered point-of-care tests<br>• Patients can also access tests via the internet, and some may receive postal invitations to test
**North East Essex PCT** (41)	North East Essex	• Home self-sampling kits for chlamydia posted to all young people (18-24years) within the PCT boundary. Tests included uniquely numbered containers to collect urine samples, instructions, information about service registration, informative material on sexual health and a prepaid envelope for kit return<br>• Individuals hoping to use the service were directed to sign up with their unique number through either a webpage or using a freephone number<br>• Individuals were notified of available results by text or other means requested and could log into their account<br>• If receiving a positive result, patients completed an online questionnaire and chose whether they would like to collect their treatment from a pharmacy or have it posted to them. A doctor remotely

		reviewed this information and prescribed treatment if appropriate.<br>• If the doctor was concerned by information provided in the questionnaire, they would contact the patient directly to discuss treatment<br>• If individuals did not have internet access they could use the freephone number to seek assistance<br>• Sexual partners could receive notification from the service and could be linked to existing cases
**Saving lives** (42)	North-West of England sexual health clinic	• OPSS service provided by a charity (Saving Lives), their partners and Public Health England Birmingham Laboratories<br>• Change of how self-samples are collected for HIV testing within this service occurred in August 2017<br>• Before August 2017, blood was collected using finger-prick capillary blood sampling into a 500µL mini-tube. From August 2017, blood was collecting using finger prick capillary blood sampling onto specialised filter paper (DBS). The volume of bloods required for DBS is much smaller than for MT.
**Sexual Health London** (43–46)	29 boroughs of London	• SHL is a consortium led by Preventx Limited, which provides the online testing service; the data controller is City of London; and the clinical governance lies with Chelsea and Westminster NHS Foundation Trust<br>• SHL was launched in 2018, and is available to residents of the participating London boroughs who are aged 16 years or older.<br>• Service users register for an account online, complete an online consultation and, if eligible, are either posted the test kit or are able to collect it from a local sexual health clinic (in some areas). Self-taken samples are posted back to the Preventx laboratory for testing.<br>• Service users with symptoms, or other needs that mean they are unsuitable for remote testing, are signposted to their local sexual health service.<br>• Local sexual health clinics refer suitable attendees to SHL.<br>• Access results via online portal (or are phone reactive HIV results)
**SH:24** (20,32,33,47–50)	Lambeth and Southwark, London;	• Individuals complete a short form on the SH:24 website to order a free postal self-sampling kit for chlamydia, gonorrhoea, HIV and syphilis; test kits for men who have sex with men (MSM) include

	Essex	rectal and pharyngeal swabs. The kits include pictorial leaflets and links to the SH:24 website which has guidance and videos of how to collect blood samples<br>• Individuals reporting symptoms are advised to visit clinics but can use SH:24 postal kits if they prefer<br>• Individuals reporting complex needs such as mental health conditions are spoken to by phone and are referred to relevant services but can still use SH:24 postal kits if they prefer<br>• Users return kits in a prepaid envelope and are sent text messages to indicate the progress of their order. SH:24 contact information is provided in these messages for individuals who would like to discuss any concerns.<br>• If SH:24 has not received the postal test within 2 weeks, a text reminder is sent and tests are resent if requested<br>• STI test results for chlamydia, gonorrhoea and syphilis are sent by text and if they are positive then patients are signposted to clinics; HIV-reactive test results are provided by telephone call.<br>• If results are positive, sexual partners can be notified
**TakeATestUK** (51)	Midlands based sexual health clinic	• OPSS service provided by a charity (Saving Lives), their partners and Public Health England Birmingham Laboratories<br>• Change of how self-samples are collected for HIV/STS testing within this service occurred in August 2017<br>• From 6th December 2016- 1st November 2017, blood was collected using finger-prick capillary blood sampling into a 500µL mini-tube. From 3rd November 2017, blood was collecting using finger prick capillary blood sampling onto specialised filter paper (DBS). The volume of bloods required for DBS is much smaller than for MT.
**Umbrella** (35,52)	Birmingham and Solihull	• Individuals self-register and fill out an online questionnaire. They are then issued a self-sampling kit, either to be posted to their chosen address or available from Umbrella pharmacies or clinics<br>• Patients with symptoms are advised to attend a face-to-face clinic, but are able to order a test online if they prefer<br>• Patients collect their samples and post the kit to a laboratory

	• Individuals are offered STI screening services if they choose to attend an Umbrella clinic<br>• If patients receive positive test results, despite which method was used for screening, they are recalled for their treatment. They receive this invitation by SMS, followed by further telephone or written contact if consent was provided
